# Selective miRNA inhibition in CD8^+^ cytotoxic T lymphocytes enhances HIV-1 specific cytotoxic responses

**DOI:** 10.3389/fimmu.2022.998368

**Published:** 2022-09-26

**Authors:** Nadia Madrid-Elena, Sergio Serrano-Villar, Carolina Gutiérrez, Beatriz Sastre, Matías Morín, Laura Luna, Laura Martín, Javier Santoyo-López, María Rosa López-Huertas, Elena Moreno, María Laura García-Bermejo, Miguel Ángel Moreno-Pelayo, Santiago Moreno

**Affiliations:** ^1^ Department of Infectious Diseases, Hospital Universitario Ramón y Cajal and Instituto de Investigación Sanitaria Ramón y Cajal (IRYCIS), Madrid, Spain; ^2^ Centro de Investigación en Red de Enfermedades Infecciosas (CIBERINFEC), Instituto de Salud Carlos III (IRYCIS), Madrid, Spain; ^3^ Department of Medicine, University of California San Francisco, San Francisco, CA, United States; ^4^ Department of Immunology, Instituto de Investigación Sanitaria (IIS)-Fundación Jiménez Díaz, Madrid, Spain; ^5^ Centro de Investigación Biomédica en Red (CIBER) de Enfermedades Respiratorias (CIBERES), Instituto de Salud Carlos III, Madrid, Spain; ^6^ Department of Genetics, Hospital Universitario Ramón y Cajal and Instituto de Investigación Sanitaria Ramón y Cajal, Madrid, Spain; ^7^ Centro de Investigación Biomédica en Red de Enfermedades Raras (CIBERER), Instituto de Salud Carlos III, Madrid, Spain; ^8^ Biomarkers and Therapeutic Targets Group and Core Facility, Instituto Ramón y Cajal de Investigación Sanitaria (Instituto de Investigación Sanitaria Ramón y Cajal), Spanish Renal Research Network (REDinREN), Madrid, Spain; ^9^ Edinburgh Genomics, The University of Edinburgh, Edinburgh, United Kingdom; ^10^ Immunopathology Unit, National Center of Microbiology, Instituto de Salud Carlos III, Madrid, Spain; ^11^ Department of Medicine, Alcalá University, Alcalá de Henares, Spain

**Keywords:** micro-RNA, cytotoxicity, cellular immunity, non-coding RNA, HIV

## Abstract

miRNAs dictate relevant virus-host interactions, offering new avenues for interventions to achieve an HIV remission. We aimed to enhance HIV-specific cytotoxic responses—a hallmark of natural HIV control— by miRNA modulation in T cells. We recruited 12 participants six elite controllers and six patients with chronic HIV infection on long-term antiretroviral therapy ("progressors"). Elite controllers exhibited stronger HIV-specific cytotoxic responses than the progressors, and their CD8+T cells showed a miRNA (hsa-miR-10a-5p) significantly downregulated. When we transfected *ex vivo* CD8^+^ T cells from progressors with a synthetic miR-10a-5p inhibitor, miR-10a-5p levels decreased in 4 out of 6 progressors, correlating with an increase in HIV-specific cytotoxic responses. The effects of miR-10a-5p inhibition on HIV-specific CTL responses were modest, short-lived, and occurred before day seven after modulation. IL-4 and TNF-α levels strongly correlated with HIV-specific cytotoxic capacity. Thus, inhibition of miR-10a-5p enhanced HIV-specific CD8^+^ T cell capacity in progressors. Our pilot study proves the concept that miRNA modulation is a feasible strategy to combat HIV persistence by enhancing specific cytotoxic immune responses, which will inform new approaches for achieving an antiretroviral therapy-free HIV remission.

## Introduction

Despite the success of antiretroviral therapy (ART) to achieve long-life HIV replication, ART results in stigma, cumulative toxicities, and a high cost to health care systems. Thus, achieving ART-free remission is a priority (1, 2). Elite controllers (ECs)—a rare subgroup of individuals showing enhanced immune control of HIV and the ability to maintain durable HIV suppression without ART— have helped understand the mechanisms that allow HIV control in the absence of ART and inspired many of the strategies under investigation in the field of HIV cure (3).

Virus-specific CD8^+^ T cells are generally recognized as the key determinant of spontaneous HIV control (2, 4, 5). Thus, approaches to enhance HIV-specific cytotoxicity are pursued as part of the response to achieve ART-free remission (1, 2). Some of the determinants generally accepted of natural HIV control include the following: (i) enrichment in some polymorphisms within the human leukocyte antigen (HLA) class I binding pocket—that would allow a more efficient presentation of HIV viral peptides to CD8^+^ T cells (6)—, (ii) higher cell-intrinsic activation of the canonical *Wnt* signalling pathway—resulting in inhibition of HIV transcription in infected cells (7)—and (iii) metabolic shifts associated to a broader polyfunctionality of HIV-specific CD8^+^ T cells (8, 9). However, the mechanisms mediating the strong HIV-specific CD8^+^ T cell responses in ECs are not well understood.

MicroRNAs (miRNAs) are small, non-coding RNA molecules that play a crucial role in modulating the translation of mRNAs into proteins (10). Besides human cells, certain viruses also code for miRNAs, which interact with the cell’s RNA interference (RNAi) machinery. Furthermore, host cellular miRNAs are also crucial for certain viruses to establish infection and may play a role in protecting against infection. Of particular interest for the field of HIV eradication, it has been shown that host miRNAs might be involved in HIV persistence in various ways, such as regulating the transition from latency to activation, the clearance of latent reservoirs, and reducing virus production (11). All these mechanisms offer an opportunity to explore new strategies to cure HIV infection.

Here, we aimed to define the miRNA signature mediating the strong HIV-specific cytotoxic T lymphocytes (CTL) response in CD8^+^ T cells from ECs, and to boost the CTL response in people who had shown progressive HIV infection after *ex vivo* modulation of a differentially expressed miRNA.

## Materials and methods

### Study participants

We included PWH from the HIV outpatient clinic of the Infectious Diseases Service of University Hospital Ramón y Cajal (Madrid, Spain). All participants gave their informed consent, and protocols were approved by the University Hospital Ramón y Cajal Ethics Committee (CEIC, ceic.hrc@salud.madrid.org).

We included 12 individuals, classified into two groups: Elite Controllers (“controllers”; n = 6) had to have confirmed HIV-1 infection with at least five years since infection and viral load <37 RNA-HIV copies/mL in the absence of ART. Chronic progressors on ART (“progressors”; n = 6) were subjects with confirmed HIV-1 infection and viral load < 37 RNA-HIV copies/mL for at least one year under stable ART. Coinfection with hepatitis C virus and active consumption of illegal drugs were not exclusion criteria. The main objective was to characterize the differential miRNA profile in a population of CD8^+^ T cells exhibiting an efficient HIV-1 specific response and a population of CD8^+^ T cells with a weak response.

In addition, samples from the six progressors were used to examine the potential role of the miRNA differentially expressed in controllers compared to progressors in the first part of the study.

### Cytolytic T lymphocytes response analysis

We used primary T cells (CD4^+^ and CD8^+^ T cells) isolated from controllers and progressors to develop an *ex vivo* assay of HIV-specific CTL response, as described previously (12). This assay tests the *ex vivo* capacity of HIV-specific CD8^+^ T cells to suppress HIV-1 superinfection of autologous CD4^+^ T cells through cytotoxic activity, this being the ultimate function of CD8^+^ T cells. Using this assay, we can differentiate effective and ineffective anti-HIV CD8^+^ T-cell responses and ineffective responses in progressors with persistent viremia.

The technique consists of three steps: a) Isolation of CD4^+^ and CD8^+^ T cells and activation of CD4^+^ T cells. Briefly, peripheral blood mononuclear cells (PBMCs) from patient blood samples were isolated using Ficoll-Hypaque density gradient (Comercial Rafer S.L., Zaragoza, Spain). The CD4^+^ T-cell fraction and the CD8^+^ T-cell fraction were isolated by positive and negative selection using CD4^+^ microbeads and CD8^+^ T Cell Isolation Kit, respectively (Miltenyi Biotech, Bergisch Gladbach, Germany). CD4^+^ T cells were cultured in RPMI 1640 medium (Gibco) supplemented with 10% (vol/vol) fetal bovine serum (FBS, Gibco), penicillin and streptomycin (1 mg/mL), and one µg/mL phytohemagglutinin-L (PHA) (Sigma-Aldrich, St. Louis, MO, USA), and IL-2 100 U/mL (Sigma-Aldrich) for three days to activate them, at 37°C in 5% CO_2_ atmosphere. CD8^+^ T cells were cultured simultaneously and under the same atmospheric conditions in RPMI medium supplemented with 10% FBS, penicillin, and streptomycin (1 mg/mL). b) *In vitro* infection of CD4^+^ T cells and CD4^+^/CD8^+^ T cell co-cultures with HIV-1. After this activation period, CD4^+^/CD8^+^ T cells co-culture was established with 10^6^ cells/mL final concentration. Superinfection was developed using HIV-1 *BaL* (CCR5-tropic virus) from the NIH AIDS Research and Reference Reagent Program. The infection dose should yield between 100 and 1000 ng/mL of HIV-1 p24 antigen at the replication peak. Usually, a multiplicity of infection (MOI) equal to 10^-3^ is enough to reach this level of infection. The virus was added to CD4^+^ T cell culture and CD4^+^/CD8^+^ T cell co-culture in a 1:1 ratio. Both cultures were maintained for 14 days at 37°C in a 5% CO_2_ atmosphere. Cell culture supernatants were harvested at 7, 10, and 14 days and frozen at -80°C until use. And c) CTL response was evaluated by quantifying p24 antigen presence in supernatants (12), using HIV-1 p24 Antigen ELISA Bulk (Zeptometrix, Buffalo, NY, USA). A decrease in p24 antigen production indicates the enhanced cytolytic activity of CD8^+^ T cells. CD4^+^ T cell culture infected *in vitro* with HIV-1 BaL and without CD8^+^ T cells was used as maximum infection control in each patient. Each sample was assayed in triplicate. CTL response was finally measured as the HIV-1 suppression activity based on p24 levels at each time point (log10 p24 decrease). For this, we calculated the difference between p24 concentrations (ng/mL) in CD4^+^ T cells HIV-1 BaL infected cultures and p24 concentrations (ng/mL) in HIV-1 BaL infected CD4^+^:CD8^+^ (1:1) co-cultures. A reduction of p24 antigen ≥ 1 log_10_ was considered a high CD8^+^ CTL response, as previously described (10). The CTL response was measured by p24 antigen on days 7, 10, and 14 post-infection.

### Identification of cellular miRNAs signatures associated with HIV-1 specific CTL response in CD8^+^ T cells

We isolated CD8^+^ T cells from controllers, progressors, and healthy subjects from PBMCs as described above. We were stored at -80°C resuspended in TRIzol Reagent until RNA extraction to massive sequencing to analyze the miRNA profile. Total RNA, preserving the small RNA fraction, was extracted using mirVana™ miRNA Isolation Kit (Life Technologies, Thermo Fisher Scientific™ Waltham, MA USA) according to the manufacturer’s indications. Ten million cells per condition were used for RNA extraction previously to next generation sequencing (NGS). The quantity and quality of the RNAs were evaluated using the Agilent 2200 TapeStation. High-throughput sequencing was carried out in a HiSeq platform (Illumina Inc.). Libraries for sequencing were prepared using TruSeq Small RNA Sample Prep Kit V1 (Illumina) following the TruSeq^®^ Small RNA Sample Preparation Guide.

Briefly, ligation of the 3´-adapter was conducted, incubating 1μg of total RNA of each sample with the adapter for 2 minutes at 70°C. Then 5´-adapter was added alongside a truncated T4-RNA ligase 2 (New England Biolabs (UK) Ltd.) in incubation at 28°C for 1 hour. Half of the ligation product was used for the reverse transcription with SuperScript II reverse transcriptase (Life Technologies, Thermo Fisher Scientific™ Waltham, MA USA) in a thermocycler for 1 hour at 50°C. Next, enrichment of the cDNA was performed using PCR cycling: 30 sec at 98°C; 11cycles of 10sec at 98°C, 30 sec at 60°C and 15 sec at 72°C; a final elongation of 10 min at 72°C, and pause at 4°C. PCR products were resolved on 6% Novex TBE PAGE gels (Life Technologies, Thermo Fisher Scientific™ Waltham, MA USA). miRNA fragments between 145 and 160bp were cut from the polyacrylamide gel and extracted using MinElute gel extraction kit (Qiagen) accordingly to manufacturers’ instructions. The libraries were visualized on an Agilent 2100 Bioanalyzer using Agilent High Sensitivity DNA kit (Agilent) and quantified using quantitative PCR with Kappa Library Quantification Kit (Master Mix and DNA Standards, Kapa Biosystems). The libraries were pooled, and 9pM pools were sequenced to generate single-ended 50bp reads. Low quality reads (Q<30) and reads containing only Illumina’s adapters were filtered out from the raw data. Filtered reads were mapped against the human hg19 reference genome using the Novoalign program that strips adapters from the reads before mapping and detects hairpin structures in the mapped region. Unique mapping reads, with a minimum length of 17 nucleotides and a maximum of one single mismatch, are obtained in a SAM file that will be sorted, indexed, and converted to a BAM file using Samtools. Total counts for miRNAs were obtained using htseq-count using a gff (gene-finding format) file with all human miRNAs features from mirBase (release 21). Normalization of miRNA counts and differential expression between two conditions was performed using DEseq2 package from Bioconductor (Liu S. et al., 2021). We then performed the dataset comparisons to obtain the differentially expressed microRNA profiles.

### Assessment of miRNA modulation/inhibition

PBMCs were obtained from progressors by Ficoll-Hypaque density gradient centrifugation. We isolated CD4^+^ T and CD8^+^ T subsets from the same PBMCs using the CD4 Microbeads and CD8^+^ T Cell Isolation Kit (Miltenyi Biotec, Bergisch Gladbach, Germany), respectively. CD4^+^ T cells were resuspended in RPMI medium supplemented with 10% FBS, penicillin and streptomycin 1 mg/ml, phytohemaglutinin-L (PHA) 1 µg/mL (Sigma-Aldrich, St. Louis, MO, USA), and IL-2 100 U/mL (Sigma-Aldrich), and incubated at 37°C in 5% CO_2_ atmosphere. CD8^+^ T cells were also cultured during the same time in RPMI supplemented with 10% FBS, penicillin and streptomycin 1 mg/ml.

CD8^+^ T cells of each patient were transfected with 100 nM of synthetic inhibitor for specific human miR-10a-5p (hsa-miR-10a-5p, miRCURY LNA Power inhibitor, Exiqon Vedbaek, Denmark), or 0.1 nM of miR-10a-5p mimic, using a Gene Pulser Electroporator (Bio-Rad Laboratories, Hercules, CA, USA). In brief, 5 million cells were collected per condition in RPMI 1640 medium without supplements and mixed. Cells were transfected in a cuvette with a 4-mm electrode gap (EquiBio) at 250 V, 950 microfarads, and maximum resistance. After transfection, cells were incubated at 37°C in 2.5 mL of supplemental RPMI, including 10% FBS, penicillin, and streptomycin 1 mg/mL. A scrambled miRNA inhibitor control expressing 5-carboxyfluorescein (FAM) as reported gene (i-scramble-miRNA-FAM) (Power inhibitor negative control, Exiqon) (100 nM) was used as control of transfection efficiency and measured by flow cytometry on a FACScalibur Flow Cytometer (BD Biosciences) using CellQuest software.

Each patient’s activated CD4^+^ T cells were co-cultured with their miRNA modulated CD8^+^ T cells transfected in a 1:1 ratio. Then, superinfection was developed using an HIV-1 BaL. The virus was added to CD4^+^ T cell culture and CD4^+^/CD8^+^ T cell co-culture. Both cultures were maintained for ten days at 37°C in a 5% CO_2_ atmosphere. Cell culture supernatants were harvested at 7, 10 days post-infection and stored at −80°C until use.

CTL response was evaluated by quantifying supernatant p24 antigen by HIV-1 p24 Antigen ELISA Bulk (Zeptometrix, Buffalo, NY, USA).

### Quantitative RT-PCR assays

We isolated total RNA containing miRNAs using mirVana miRNA Isolation Kit (Life Technologies, Thermo Fisher Scientific™ Waltham, MA USA), and we synthesized cDNA from 100ng of total RNA by using the miRCURY LNA Universal RT microRNA PCR System (Exiqon), according to the manufacturer’s instructions. cDNA synthesis was performed in the following conditions: 42°C, 60 min; 95°C, 5 min. Expression of miR-10a-5p was quantified using a specific miRNA-LNA™ PCR primer set for PCR amplification purchased from Exiqon.

The small 5S rRNA was used as internal controls for data normalization with a 5S rRNA PCR primer set (Exiqon). ExiLENT SYBR Green PCR Master Mix (Exiqon) was used according to the manufacturer’s instructions. PCR cycle conditions were 95°C, 10 min; 45 cycles: 95°C, 10 sec; 60°C, 1 min; and melting curve analysis. All reactions were performed in a LightCycler 480 Real-Time PCR System (Roche Diagnostics). Cel-miR-39-3p was used as an RT control to validate the efficiency of RNA extraction. Cts observed for all the miRNAs were lower than 35, although miRNAs inhibition renders Higher Ct values as expected. Data analysis was performed using the second derivative method since we confirm the efficiency of the qRT-PCR as 2. Once the threshold cycle (Ct) was obtained, data were normalized with the Ct values of 5S rRNA and analyzed with the formula 2^-ΔΔCt,^ where mock-transfected cells are considered a reference control. The scrambled condition was also used as the control group for analyzing data.

### Cytokine and cytotoxicity markers assessment

For the assessment of cytokines and cytotoxicity markers, we performed multi-analyte profiling using the ProcartaPlex immunoassay (TermoFisher Scientific) used in combination with the Luminex instrument platform (MagPix, Luminex Corporation). The panel measured as cytokine anti-inflammatory: interleukin-4 (IL-4) and interleukin-10 (IL-10); pro-inflammatory: interleukin-2 (IL-2), interleukin-6 (IL-6), interleukin-17A (IL17A), IFN-gamma and tumor necrosis factor-alpha (TNF-alpha); and cytotoxicity markers: granzyme A, granzyme B, and perforin. The assay was conducted according to the manufacturer’s instructions. All cytokine and cytotoxicity markers from a single patient were analyzed in one run.

### Correlation analyses

We used R software for the correlation analyses for cytolytic activity, cytokine, and cytotoxicity markers measured concomitantly at days 7 and 10 (library corrplot).

### *In silico* prediction of specific HIV related functions of miR-10a-5p

**Figure 1:**
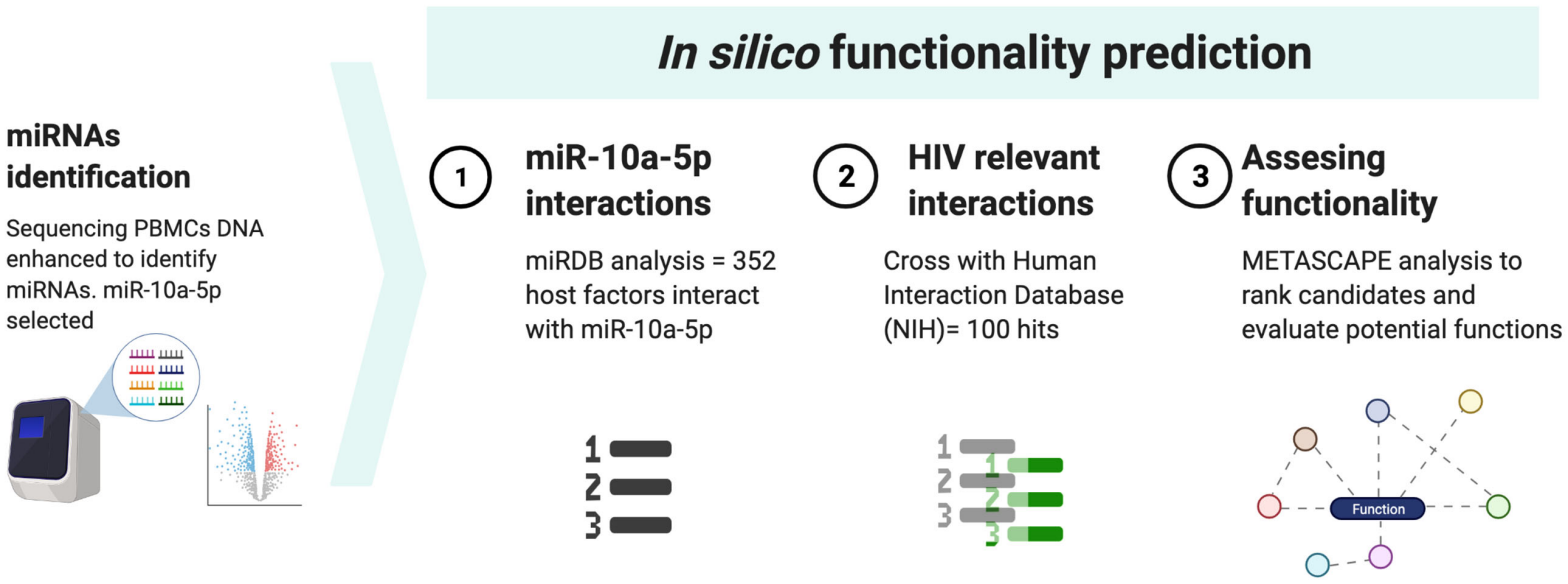
Workflow for *in silico* prediction of miR-10a-5p.

To further evaluate the potential role of the identified miRNA as a booster of HIV-specific CTL capacity we explored a database specific for miRNA expression (miRDB) (13). Then the identified mRNAs candidates exhibiting interactions with this miRNA were crossed with the whole list of host-pathogens interactions publicly available at NIH website (Human Interaction Database) (14) to identify specific functions related with HIV. Then, a GO-based enrichment analysis was performed in Metascape web tool (15) to evaluate biological functions of these candidates. We obtained a list with all the cellular functions in general and another list only selected the HIV-related functions and ordered according to the p-value obtained in the GO-based analysis (**Table S2**). The p-value evaluates significant presence; therefore we selected those factors that have a p-value below -4 (the more negative, the better) to obtain the most relevant. The methods are summarized in **Figure 1**.

### Data availability

The sequencing data for this study have been deposited in the European Nucleotide Archive (ENA) at EMBL-EBI under accession number PRJEB55140 (https://www.ebi.ac.uk/ena/browser/view/PRJEB55140).

## Results

### Characteristics of the study population

We included 12 individuals: 6 Elite controllers (“controllers”) and six individuals with chronic HIV infection on long-term ART (“progressors”). Their clinical and demographic characteristics are shown in **Tables 1**, **S1**. All patients were male, with ages ranging from 35 to 57 years. The frequency of past history of injection drug uses was higher in progressors (50%) than in controllers (0%). CD4 cell counts at study entry were higher in progressors (median [IQR], 836 [584-1114] cells/mm^3^) than in the EC group (639 [488-1016] cells/mm^3^). The median nadir CD4^+^ T-cell count was 496 cells/mm^3^ in controllers and 553 cells/mm^3^ in progressors. All patients had plasma HIV-1 RNA <1.57 log_10_ copies/ml. The median time since the diagnosis of HIV ranged from 10 to 14 years. The median duration of ART before study entry in progressors was 90 months.

**Table 1:** General characteristics of study participants.

	Controllers (n = 6)	Progressors (n = 6)
**Male gender (%)**	6 (100%)	6 (100%)
**Age, years (median [IQR])**	55 [41.75-57]	47 [41-48]
**MSM (%)**	1 (17%)	5 (83%)
**Heterosexual (%)**	2 (33%)	0 (0%)
**Past injection drug use (%)**	3 (50%)	0 (0%)
**CD4^+^ T-cell count (cells/mm^3^) (median [IQR])**	639 [488-1016]	836 [584-1114]
**Nadir CD4^+^ T-cell count (cells/mm^3^) (median [IQR])**	496 [292-803]	553 [208-668]
**CD8^+^ T-cell count (cells/mm^3^) (median [IQR])**	813 [533-967]	1014 [699-1279]
**HIV-1 RNA (log10 copies/mL)**	<1.57	<1.57
**Time since HIV diagnosis, years** **(median [IQR])**	14 [5-27]	10 [5-17]
**ART duration (months [IQR])**	-	90 [51-153]

-, Non applicable.

### Characterization of HIV-specific CD8^+^ T cells responses

**Figure 2:**
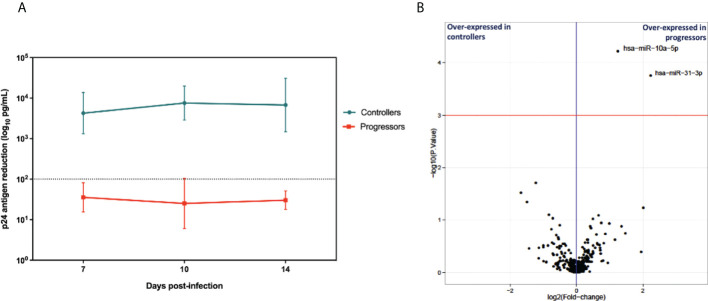
**(A)** Cytolytic activity of CD8^+^ T cells from EC and CP evaluated by p24 antigen quantification (n=6 in each group). Depicts the mean and stand deviation of p24 (ng/ml) levels for each patient at the different time points. HIV-suppressive capacity of CD8^+^ T cells is calculated at the peak of viral replication in CD4^+^ T cells alone, as the log decrease in p24 production when CD4^+^ T cells are cocultured with CD8^+^ T cells. Experiments run in triplicate. **(B)** Volcano plot depicting log2 of mean fold changes in miRNA expression (X-axis) and -log10 P values (Y-axis).

To identify the miRNAs signature associated with HIV-1 specific CTL response, we first evaluated the CTL response in each group. Then, we developed an *ex vivo* model using primary CD4^+^ and CD8^+^ T cells to assess the HIV-specific CTL, which was measured on days 7, 10, and 14. The CD8^+^ T cells from controllers exhibited an efficient suppressive capacity, measured as the >2 log reduction of p24 levels in co-cultures supernatants (**Figure 2A**). In contrast, all progressors showed a suppressive ability below two logs.

### Identification of cellular miRNAs signatures associated with HIV-1 specific CTL response in CD8^+^ T cells

To identify cellular miRNAs signatures associated with HIV-1 specific CTL response hen, we compared differentially expressed miRNAs. We used the total RNA extracted from the infected CD8^+^ T cells from each group, preserving the small RNA fraction to measure miRNA expression using next-generation sequencing. Changes in miRNAs expression were considered significant when the normalized mean counts per million (CPMs) across all samples was higher than 10 CPMs, the fold change (progressor/controller) was >1 (upregulated miRNAs) or <1 (downregulated miRNAs), the p-value was <0.001 and the adjusted p-value (false discovery rate [FDR]) was <0.05. Two microRNAs were upregulated in the CD8+ T cells from progressors, miR10a-5p and miR-31-3p. They fulfilled the statistical criteria, but only miR10a-5p mean values were above 10 CPMs in all samples, and thus it was selected for further experiments (**Table S2** and **Figure 2B**).

### *In silico* prediction of specific HIV-related functions of miR-10a-5p

To evaluate the potential role of the identified miRNA as a booster of HIV-specific CTL capacity we explored the predicted mRNA targets implicated in host-virus interactions. This analysis revealed that miR-10a-5p could regulate the expression of several proteins involved in direct interactions with HIV-1 proteins, and in relevant immune functions including antigenicity, cell cycle, chemotaxis and natural immunity. For example, among the most relevant hits we found that miR-10a-5p modulates the expression of transferrin receptor protein 1 (TFRC), which interacts with the HIV-1 proteins gp120, gag, nef, tat, vif and vpu, implicated in viral entry and antagonism of cell restriction factors (16). Key proteins for immune cell chemotaxis such as syndecan-1 (SDC1) or gamma actin (ACTG1) are also predicted targets. Interestingly, a calcium-dependent protein kinase (CAMK2B) implicated in the activation of natural immune response against HIV *via* NK activation, a hallmark of spontaneous HIV control (17), has been identified as a miR-10a-5p relevant target (18). Remarkably, CAMK2B is also is inhibited by the HIV-1 tat protein but also a miR-10a-5p relevant target.

**Table 2** shows a selection of the most relevant miR-10a-5p targets. Detailed results can be found in **Table S3**.

**Table 2:** Selection of most relevant miR-10a-5p targets involved in relevant host-HIV interactions.

Genes	Protein name	LogP	HIV-related function or interaction
**TFRC**	Transferrin receptor protein 1 isoform 1	-7,94	Interacts with gp120, gag, nef, tat, vif and vpu.
**XRN1**	5’-3’ exoribonuclease 1 isoform a	-7,94	Present in HIV-1 Gag virus-like particles.
**NCOR2**	Nuclear receptor corepressor 2 (NCOR2)	-7,22	NCOR2 knockdown by siRNA inhibits HIV-1 replication.
**BACH2**	Transcription regulator protein BACH2	-6,97	BACH2 gene favors HIV-1 integration for expansion and persistence of infected cells.
**TRIM66**	Tripartite motif containing 66	-6,85	TRIM66 knowckdown by siRNA inhibits the early stages of HIV-1 replication
**SDC1**	Syndecan-1 precursor	-6,55	Binds Tat and gp120. HIV-1 Tat tethered to the surface of syndecan-1 expression promotes PBMCs transendothelial migration. SDC1 knockdown by siRNA inhibits HIV-1 replication.
**ACTG1**	Actin, cytoplasmic 2	-6,41	HIV-1 Nef inhibits CXCL12 induced chemotaxis, monocytes, and PBMCs, which leads to marked downregulation of F-actin accumulation in cells. Actin is hydrolyzed by HIV-1 protease during acute infection.
**CREB1**	Cyclic AMP-responsive element-binding protein 1 isoform A	-6,21	CREB1 phosphorilation induces several processes related with gp120, MA, Tat and Vpr proteins of HIV. i.e.: extracellular HIV-1 Tat protein induces the rapid activation of CREB transcription factor through a signal cascade involving the MAPK pathway.
**MAP3K7**	Mitogen-activated protein kinase kinase kinase 7 isoform A	-5,67	Several functions related to viral cycle. Interactions with viral proteins, such as HIV-1 Nef, resulting in MAP3K7 activation in M2-macrophages. Mediates HIV-1 Nef-induced strong activation *via* MAP kinases and NF-kappaB pathway in M2-macrophages.
**CAMK2B**	Calcium/calmodulin-dependent protein kinase type II subunit beta isoform 1	-5,37	HIV-1 Tat inhibits the activation of CAMKII in NK cells, providing evidence that Tat inhibits NK cell activation which might contribute to the impairment of natural immunity in HIV-1 infection. Tat-induced IL-10 expression is regulated by p38 MAPK- and CaMK II-activated CREB-1 as well as Sp-1 transcription factors
**FXR1**	Fragile X mental retardation syndrome-related protein 1 isoform A	-4,67	FXR1interacts with HIV-1 Tat in HeLa cells and HIV-1 Gag. Knockdown of FXR1 by siRNA inhibits the early stages of HIV-1 replication.
**RB1CC1**	RB1-inducible coiled-coil 1	-4,43	Knockdown of RB1CC1 by siRNA inhibits HIV-1 replication in HeLa P4/R5 cells

LogP, Log10(P-value), of the GO-based enrichment analysis performed in Metascape (applies the standard accumulative hypergeometric statistical test to identify ontology terms, where input genes show significant presence).

### Inhibition of miR10a-5p enhances CTL response of CD8^+^ T cells from chronic progressors under ART

**Figure 3:**
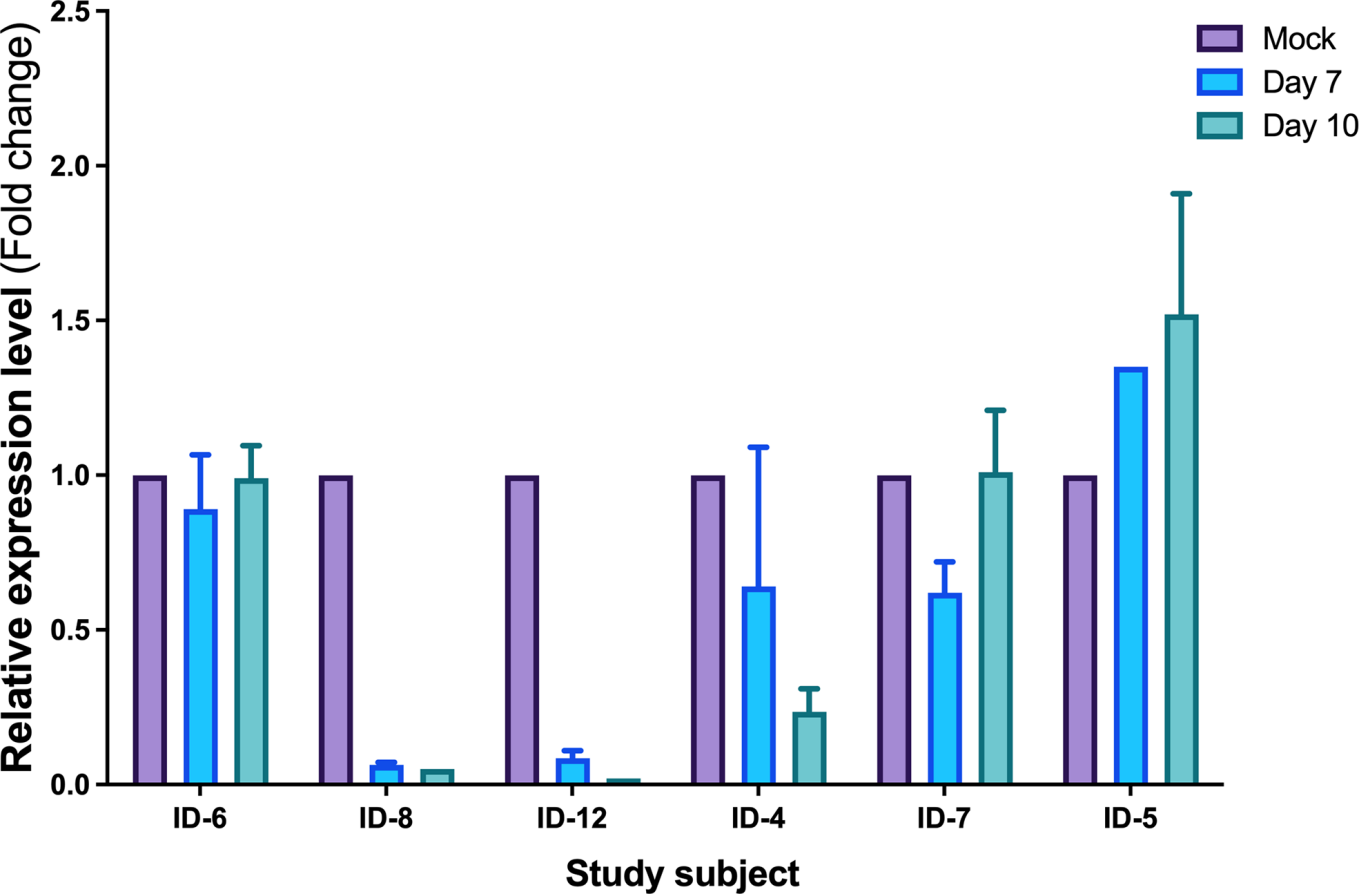
Expression levels of miR-10a-5p measured by qRT-PCR after modulation. Data are the mean ± SEM and are expressed as fold change using 5S levels as the reference (2−ΔΔCt). Purple histograms represent the mean of expression values from 5S, blue histograms represent the mean of expression values from day 7 and green from day 10. Expression values are normalized to the value of the control condition. Error bars represent the standard error of the mean of the three relative expressions from three biological replicates and two technical duplicates of each treatment.

To establish the functional implications of miR-10a-5p in the CTL response, we transfected CD8^+^ T cells from progressors using a specific miR-10a-5p inhibitor and appropriate controls. First, we verified the transfection efficacy of miR-10a-5p inhibitor by measuring the expression in total RNA from the HIV-infected CD8^+^ T cells using qRT-PCR. When we measured miR-10a-5p expression by qPCR at days 7 and 10 in CD8^+^ T cells *ex vivo*, we found that at day seven, miR-10a-5p levels decreased in 4 out of 6 cases (**Figure 3**).

**Figure 4:**
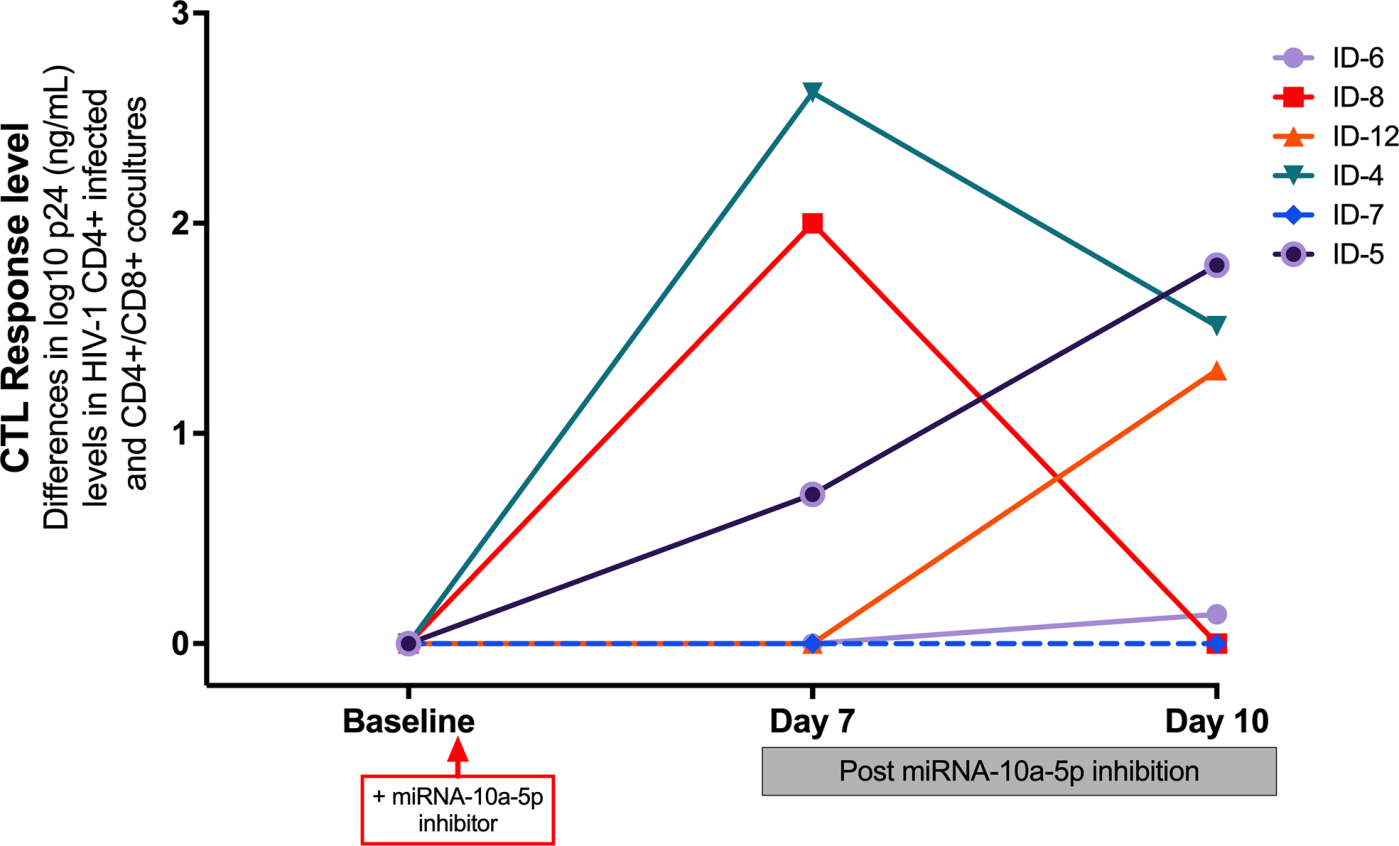
Impact of transfected miR-10a-5p inhibitor in CD8^+^ T cells from progressors at day 7 and at day 10 post-treatment on CTL response level, measured as the reduction in log10 p24 levels at baseline and at days 7 and 10 after miRNA-10a-5p inhibition. A reduction of p24 antigen ≥ 1 log_10_ was considered a relevant increase of CD8^+^ CTL response. For each experiment, we used three biological replicates and two technical duplicates.

Remarkably, we found that the most significant increases of CTL activity occurred in those experiments where miR10a-5p inhibition was more potent (subjects 4, 8 and 12), indicating that inhibition of miR10a-5p enhanced HIV-specific CTL response in progressors (**Figure 4**)

Since HIV-specific CD8^+^ T cells from HIV viremic individuals generally inhibit HIV CD4+ T cell infection in less than 1 log of p24 assayed by ELISA (12, 19), we arbitrarily considered an effective CTL activity enhancement after miRNAs modulation if CD8^+^ T cells could reduce p24 antigen levels by more than one log. Because the peak of viral replication when using the p24 ELISA assay would generally be observed on day 7 or 10 after miR-10a-5p modulation, we measured the reduction of p24 up to day ten after treatment with miR-10a-5p inhibitor. While we did not observe viral inhibition activity from CD8^+^ T cells modified with miR-10a-5p inhibitor through day 10 in two out of six experiments, increases of HIV-specific responses were apparent in the remaining four progressors, with a reduction in p24 levels between 0.71–2.622 logs. As shown in **Figure 3** miR-10a-5p inhibition of CD8^+^ T cells reduced p24 expression by ≥2 logs in two instances at day 7, which was not maintained at day 10. We found a p24 reduction of less than 2 logs in the other two cases at day 10. We did not observe significant decreases in p24 expression (i.e., ≥ 1 log p24 reduction) through day 10 in any case. The peak of viral replication in modified CD8^+^ T cell with miR-10a-5p inhibitor was 2.622 and 1.8 log_10_ at days seven and 10, respectively. These results indicate that miR-10a-5p might modestly influence CD8^+^ T cell cytotoxic activity. The effects of miR-10a-5p inhibition on HIV-specific CTL responses were short-lived, occurring before day seven after modulation, with variable outcomes through day 10.

### Dynamics inflammatory cytokines and cytotoxicity markers after miRNAs modulation

**Figure 5:**
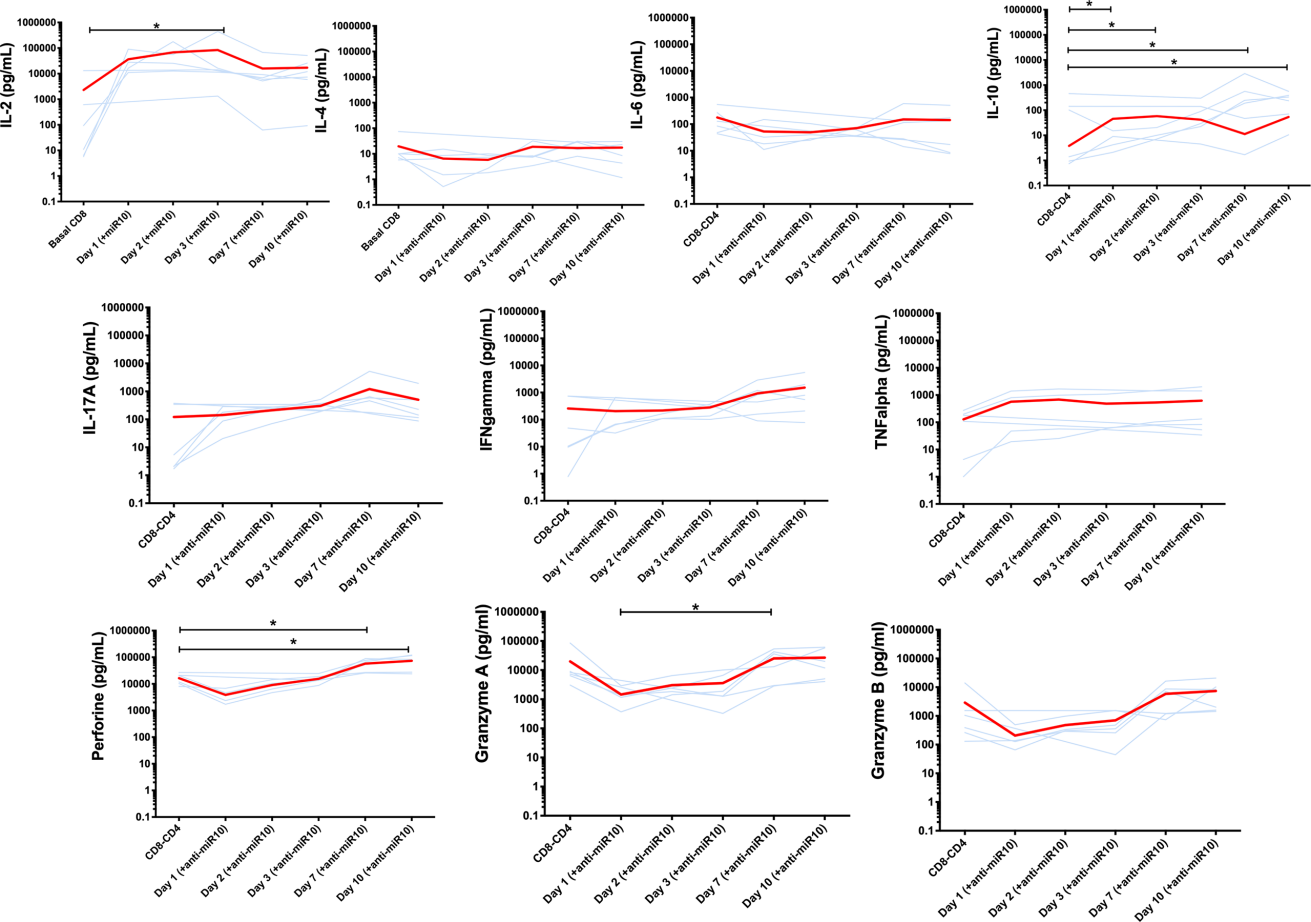
Cytokines secretion and cytotoxicity markers production before and after transfection of CD8^+^ T cells from progressors with miR-10a-5p inhibitor. Light blue lines represent individual trajectories and red lines the mean trajectories for IL-2, IL-4, IL-6, IL-10, IL-17A, IFNg, TNFa, perforin, granzyme A and B levels from three biological triplicates and two technical duplicates. Significant changes between time points were determined using a Wilcoxon sign-rank test. *p<0.05.

To further understand the potential role of miR-10a-5p on the cytotoxic capacity of CD8^+^ T cells, we measured several soluble proinflammatory and immunoregulatory cytokines before and after transfection of CD8^+^ T cells from progressors with miR-10a-5p inhibitor. Interleukin (IL) 2, IL4, IL6, IL10, IL17a, interferon (IFN) γ, tumor necrosis factor (TNF) α, as well as cytotoxicity markers including granzyme A, granzyme B, and perforin, at 0, 24 hours, 48 hours, 72 hours, seven days and ten days after treatment with miR-10a-5p inhibitor were measured by Luminex (**Figure 5**).

We found a similar pattern of IL-4 and IL-10 changes, with a progressive decay from baseline until 72 hours after miR-10a-5p inhibition and a slight increase after day 3. These changes were more apparent for IL-10. In two cases, IL-10 levels were not detectable on days 1 and 2 after modulation with anti-miRNA10.

Regarding the changes of pro-inflammatory cytokines following miR-10a-5p inhibition, IL-2 and TNF-α were those that more rapidly increased, peaking at day 3. For IL-17A, we found a less marked increase through day 7 (p=ns.), while for IFNγ, we did not appreciate a clear pattern after miR-10a-5p inhibitor treatment in progressors, but no significant differences were noted. Finally, IL-6 tended to decrease from baseline and then return to baseline levels after day 7 (p=ns.)

The cytotoxicity markers granzyme A, granzyme B, and perforin showed a consistent pattern of changes, with some of the between time-point comparisons reaching the threshold of statistical significance. All three biomarkers gradually decreased from baseline through day 3, subsequently increasing above baseline levels through day 10 (**Figure 5**).

### Links between inflammatory cytokines, cytotoxicity, and HIV-specific cytotoxicity following miR-10a-5p inhibition in progressors

**Figure 6:**
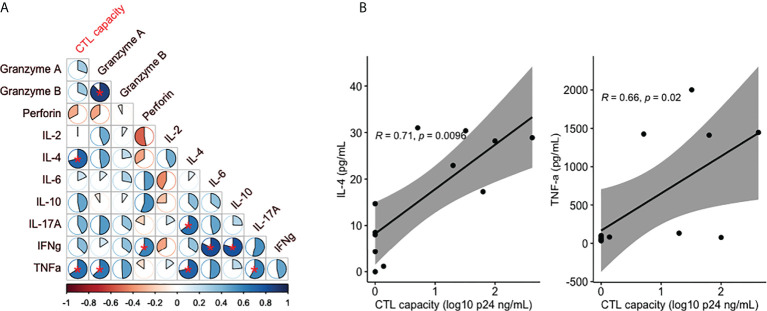
**(A)** Heatmap of correlations between cytolytic activity, cytokines and cytotoxicity markers. The pie charts depict the magnitude of each individual Spearman Rho correlation coefficient in a color gradient from red (Rho -1) to blue (Rho +1). Correlations with a P<0.05 are marked with a red asterisk. **(B)** Correlation analysis for those variables significantly associated with CTL capacity.

To obtain an overall view of the links between changes in levels of inflammatory and cytotoxicity markers and HIV-specific CTL responses following miR-10a-5p inhibition, we illustrated their correlations using all measurements (**Figure 6A**). IL-4 and TNF-α showed strong direct correlations with HIV-specific CTL capacity (Rho= 0.71, P = 0.0096 and Rho= 0.66, P = 0.02, respectively; **Figure 6B**).

## Discussion

In this pilot study, we aimed to identify cellular miRNAs signatures associated with HIV-1 specific CTL response in CD8^+^ T cells that could be modulated as part of a strategy for a functional HIV cure. We found that a single miRNA, miR-10a-5p, was differentially expressed in elite controllers and progressors. Furthermore, the *ex vivo* miRNA modulation in ART-treated progressors resulted in a modest and variable improvement in the HIV-specific cytotoxic activity.

The role of miRNAs in HIV persistence have been explored before. Specifically, specific cellular miRNAs can regulate directly (11, 20–22), and indirectly (23–27) viral gene expression, thereby affecting viral infection. The viral 3’UTR is common for all viral transcripts and presents conserved sites for all five anti-HIV cellular miRNAs identified to bind and regulate viral latency by Huang and col (11). Similarly, our group explored this possibility, but we could not identify miRNAs that could be successfully manipulated to break HIV latency in resting CD4^+^ T cells (28).

Another interesting strategy consists of restoring the killing capacity of cells by miRNA modulation. Therefore, the involvement of miRNAs in CD8^+^ T cells cytotoxicity has also been investigated (29–35). However, to our knowledge, no attempts have been made to restore the killing capacity of cells by miRNA modulation. While ART may recover to some extent the effector functions of HIV-specific CTLs, the number of these circulating cells remains reduced, and their capacity to lyse infected cells is lower than the observed in elite controllers (36). Trifari and colleagues identified that miR-139, miR-150, and, to a lesser degree, miR-342 were components of a post-transcriptional regulatory mechanism that controls the differentiation of CTLs, in response to IL-2 and other inflammatory signals (37). Hence, differential miRNA profiles between PWH at different stages of disease progression could reveal a dysregulated miRNA pattern with a prognostic and diagnostic value for HIV-1 therapies. Thus, differential regulation of miRNAs in CD8^+^ T-lymphocytes could help us better understand the underlying mechanisms of an effective HIV antiviral response that controls viral replication and may predict disease progression status. Moreover, identification of miRNAs involved in CD8^+^ T cell cytotoxic activity could lead to develop efficient approaches for HIV control by ex vivo modified T cells, analogously to tumour control by CART cells or NK cells therapies.

The miRNA expression profile in CD8^+^ T cells from HIV-infected individuals and its relationship with viral replication and effective antiviral immune responses has been assessed before (30). The miRNA-10a has been identified in some CRISPR high-throughput analysis in different infected cells with diverse types of viruses (SARS-Cov-2 and other coronaviruses, HCV, HAV, Ebola, Enterovirus 68, etc) limiting viral production (38–41). Regarding specifically to HIV persistency, Reynoso and colleagues (32) found that some miRNAs correlate to the duration of HIV infection, independently of elite control, supporting the idea that the stage of HIV disease and the immunopathogenic mechanisms driving a better spontaneous control could be linked to a specific miRNA signature. There is, therefore, the possibility that specific miRNAs can be identified and manipulated to enhance HIV-specific CTL responses. The predominant group of individuals who exhibit impaired HIV-specific CTL responses, represented in our study by the progressors, would be a target population to treat with a specific miRNA inhibitor to boost the HIV-specific CTL response. Our results indicate that a single miRNA, miR-10a-5p, is a candidate target to boost HIV-specific CTL responses.

The modulation of a single miRNA could affect CTL activity and the production of cytokines by CD8^+^ T cells, which could impact both HIV replication and latency, highlighting miR10a-5p modulation as a potential therapeutic approach in HIV eradication. Other studies have previously implicated miR-10a-5p in HIV immunopathogenesis (42, 43), but its role in cytotoxic response was unknown. Our *ex vivo* studies suggest that downregulating the expression of miR-10a-5p in CD8^+^ T cells from patients with HIV-1 infection may become a strategy to target HIV infection. Although we observed inter-individual variability, we appreciated a significant improvement in the control of HIV replication in patients with chronic, progressive disease under stable ART. Some of them reached the level of HIV-specific CTL responses seen in elite controllers. In this study, the reduction of HIV replication occurred after seven days of modification with a miR-10a-5p inhibitor in more than 50% of patients. However, it was not appreciated at day 10, indicating that the effect of CD8^+^ T cell transfection with the miRNA inhibitor was short-lived.

To further explore the mechanisms involved in the enhanced CD8^+^ cytotoxicity by miR10-5p inhibition, we determined the production of cytokines and cytotoxicity markers of modified CD8^+^ T cells from patients with a weak response. Although we lacked statistical power to test the trajectories of these biomarkers, miR-10a-5p inhibition led to an increase in IL-2, IL-17A, and TNF-α concentrations 24 h after modulation. The expression of IL-4, IL-6, IL-10, IFN-γ, perforin, granzyme B, and granzyme B was not affected by miR-10 until day 2. These results suggest that inhibition of miR-10a-5p involves cytokine and cytotoxicity markers production modulation might be contributing to the enhanced cytotoxic activity. Furthermore, we noticed that the production of cytokines and cytotoxicity markers by CD8^+^ T cells after miR-10a-5p modulation might be consistent with the cytokine pattern of elite controllers. HIV-specific CTLs in controllers contain a high proportion of multi-functional effector cells, capable of secreting TNF-α and IL-2 together with IFN-γ and show greater proliferation after antigenic stimuli than in patients with chronic progressive infection (44, 45).

Importantly, we observed a positive correlation between two cytokines (TNF-α and IL-4) and CTL in progressors-modified CD8^+^ T cells. Increased TNF-α production is a hallmark of elite control (46, 47). Furthermore, the cytokine secretion of TNF-α has been implicated in the controllers’ ability to suppress virological replication due to their cytotoxic activity against infected cells (48). In addition, IL-4 levels in controllers are twice as high as those of ART-treated patients and people without HIV. On the other hand, IL-4 can increase HIV replication by modifying viral infectivity and sensitivity to TRIM5α. These findings suggest that miR10-5p inhibition in CD8^+^ T cells may promote HIV-specific CTL responses *via* IL-4 and TNF-α signalling, in keep with previous observations in elite controllers. However, the fact that in our study IL-4 levels were not affected by miR-10a-5p inhibition but correlated with HIV-specific cytotoxicity suggests that IL-4 is implicated in the CD8^+^ T cell to ability to decrease HIV replication through mechanisms non-targeted by miR-10a-5p. Last CD8^+^ T cells from most controllers showed a rapid *de novo* expression of perforin, a mechanism associated with HIV inhibition *ex vivo* (19). However, whether these proinflammatory and anti-inflammatory cytokines are the direct targets of miR-10a-5p remains unclear, although this miRNA has been extensively linked to inflammation regulation in several settings (49). Furthermore, our *in silico* analysis of miRNA-10-5p targets related to HIV infection contributes to the field with a list of relevant candidates for further studies. Several of these factors are involved in processes such as cytokine signalling, T-cell receptor signalling, TNF signalling and other functions related to viral cycle (through direct interactions with several viral proteins), that could potentially be involved in the CTL response to HIV infection mediated by miR-10-5p.

The main strength or our work is the novelty and the potential implementation of the approach in clinical practice, modulating CD8^+^ T cells ex vivo. We were able to demonstrate that it is possible to enhance HIV-specific cytotoxicity by manipulating CD8^+^ T cells using a synthetic miRNA inhibitor. Our study is also subject to several limitations. First, we did not characterize the mechanisms by which miR-10a-5p inhibition increased CTL responses. Second, due to the limited sample size, we were unable to assess the determinates of the variability in the response assessed in our study. We are planning to assess both limitations in a next study aimed at reproducing these results. Third, ECs are now considered an heterogenous population. Fourth, cell-sorting would have improved our understanding of the CD8^+^ T-cell subsets more likely to drive the effects. However, the volume of blood required for untargeted miRNA NGS identification exceeded the maximal amount authorized by our Ethics Committee. Exploring miR-10a-5p expression levels in sorted CD8+ T-cell memory subsets will be necessary for a better biological interpretation of our results. Last, the effect on HIV-specific cytotoxic responses achieved after miR-10a-5p inhibition was transient, and could be enhanced by other methods facilitating a more sustained inhibition over time. Further studies should focus on the even rarer phenotype of individuals with exceptionally long natural HIV control to account for the variability in the underlying mechanisms driving long-term control.

In summary, in this proof-of-concept study we have identified that inhibition of a miR-10a-5p can enhance CD8^+^ T cell cytotoxicity *ex vivo* in patients with chronic HIV-1 infection on long-term ART. Although miR-10 inhibition resulted in increased HIV-specific CTL responses, the effect was modest, short-lived and variable across patients. Larger studies are required to validate the effects of miR-10a-5p and evaluate the role of modulating this miRNA to achieve an HIV functional cure. miRNA modulation is a promising approach to boost HIV-specific immunity.

## Data availability statement

The sequencing data presented in this study are deposited in the European Nucleotide Archive (ENA) repository at EMBL-EBI under accession number PRJEB55140 (https://www.ebi.ac.uk/ena/browser/view/PRJEB55140).

## Ethics statement

The studies involving human participants were reviewed and approved by Hospital Ramón y Cajal. The patients provided their written informed consent to participate in this study.

## Author contributions

Study conceptualization, SM, SS-V, MG-B, MA-P. Methodology, N-ME, SS-V, CG, BS, MM, ML-H, JS-L, EM, MG-B, MA-P, SM. Patient recruitment, SS-V, SM. Laboratory measurements, N-ME, CG, BS, MM, LL, LM, ML-H, MG-B, MA-P. Visualization, N-ME, SS-V, MM, JS-L. Supervision, MG-B, MA-P, SS-V. Writing the original draft, N-ME and SS-V. Review, editing and approval of manuscript N-ME, SS-V, CG, BS, MM, LL, LM, JS-L, EM, ML-H, MG-B, MA-P, SM.

## Funding

This research was funded by Instituto de Salud Carlos III (PIE13/00040, AC17/00019, PI18/0267, PI18/00154, PI20/00945, PI21/00141, and the TRANSCAN-2 program, European Development Regional Fund and A way to achieve Europe (ERDF).

## Acknowledgments

This study would not have been possible without the collaboration of all the patients, medical and nursery staff and data managers who have taken part in the project. **Figure 1** was generated in www.biorender.com.

## Conflict of interest

Outside the submitted work, author SS-V reports personal fees from ViiV Healthcare, Janssen Cilag, Gilead Sciences, and MSD as well as non-financial support from ViiV Healthcare and Gilead Sciences and research grants from MSD and Gilead Sciences. Author SM reports grants, personal fees and non-financial support from ViiV Healthcare, personal fees and non-financial support from Janssen, grants, personal fees and non-financial support from MSD, grants, personal fees and non-financial support from Gilead.

The remaining authors declare that the research was conducted in the absence of any commercial or financial relationships that could be construed as a potential conflict of interest.

## Publisher’s note

All claims expressed in this article are solely those of the authors and do not necessarily represent those of their affiliated organizations, or those of the publisher, the editors and the reviewers. Any product that may be evaluated in this article, or claim that may be made by its manufacturer, is not guaranteed or endorsed by the publisher.
